# The Prognostic Value of Peak Cardiac Power Output in Chinese Patients with Chronic Heart Failure

**DOI:** 10.1371/journal.pone.0147423

**Published:** 2016-01-25

**Authors:** Yuqin Shen, Haoming Song, Wenlin Ma, Zhu Gong, Yi Ni, Xiaoyu Zhang, Wenjun Xu, Jinfa Jiang, Lin Che, Jiahong Xu, Wenwen Yan, Lin Zhou, Guanghe Li, Qiping Zhang, Lemin Wang

**Affiliations:** 1 Department of Cardiology, Tongji Hospital of Tongji University, Shanghai, China; 2 Department of Rheumatology, Tongji Hospital of Tongji University, Shanghai, China; University of Naples Federico II, ITALY

## Abstract

**Background:**

Cardiopulmonary exercise testing has been widely used to risk stratify patients with chronic heart failure (CHF). Peak oxygen consumption (peakVO_2_) was regarded as a powerful predictor of survival, as it is a surrogate for peak cardiac output (CO), which by most is considered the “true” measure of heart failure. Therefore, it is reasonable to hypothesize that CO is an even stronger predictor than peak VO_2_. The present study is aimed to investigate the prognostic value of peak cardiac power output (peak CPO) in comparison with peakVO_2_ in Chinese patients with CHF.

**Methods:**

Participants provided written informed consent to participate in this study. Totally 129 patients with CHF underwent symptom-limited cardiopulmonary exercise testing (CPET), with mean age 59.1±11.4 years, 87.6% male, 57.4% ischemic etiology, body mass index (BMI) 24.7±3.7 kg/m^2^ and LVEF 38±9%. CO was measured using an inert gas rebreathing method. The primary endpoints are cardiac deaths.

**Results:**

Over median 33.7-month follow-up, 19 cardiac deaths were reported. Among peak VO_2_,VE/VCO_2_ slope and Peak CPO, their area under ROC were 0.64, 0.67, 0.68, respectively (*Ρ*<0.05).The optimal thresholds for predicting cardiac deaths were peak VO_2_≤13.4 ml.kg^-1^.min^-1^, and VE/VCO_2_ slope≥39.3 and peak CPO≤ 1.1 respectively by ROC analysis. Finally, in patients with a peak VO2≤13.4 ml.kg^-1^.min^-1^ those with peak CPO>1.1W had better survival than those with peak CPO ≤ 1.1W. However, by multivariate analysis adjusted for age, sex, BMI, resting heart rate, LVMI, LVEF, Peak CPO was not an independent predictor of cardiac deaths (*P*> 0.05).

**Conclusions:**

Peak CPO was not a predictor of cardiac death in Chinese CHF patients.

## Introduction

Persistently low cardiac output (CO) is associated with longer hospital stay, increasing morbidity and mortality. Therefore, CO during exercise has been used as an important risk predictor. Indicator dilution is a standard technique to measure cardiac output. However, this technique is not only invasive and expensive, but also can cause many complications such as bleedings, pneumothorax, arrhythmias, infection and catheter dislodgement. That is why peak oxygen consumption (peakVO_2_) as a surrogate for peak cardiac output has been widely used as a powerful predictor of survival. In recent years, some noninvasive methods to measure CO have been developed. Inert gas rebreathing by cardiopulmonary exercise testing (CPET) is a novel noninvasive method to measure CO during exercise, which is a safe, reliable, and easily performed in patients with CHF [1].Peak cardiac power output (peak CPO), a derived variable of the peak mean arterial blood pressure and peak CO [2], has emerged as a powerful independent predictor of prognosis in CHF patients. Beyond CO and peak CPO, there are other useful variables from CPET for prognosis, such as peak VO_2_ and the ventilation (VE)/carbon dioxide production (VCO_2_) slope.

The estimated prevalence of heart failure is 0.9% in China, there are approximately 4 million patients with CHF, with the number increasing annually [3].High morbidity and mortality as well as recurrent hospitalizations due to CHF have become increasing global challenge. The present study was aimed at investigating the prognostic value of peak CPO by inert gas rebreathing for cardiac-related mortality in Chinese CHF patients in comparison with peak VO_2_ and VE/VCO_2_ slope.

## Materials and Methods

### Patients and Ethical Statement

From August 2007 and May 2013, CHF patients were recruited prospectively at the Department of Cardiology of affiliated Tongji Hospital of Tongji University after signing informed consent. All of the experiments were approved by and followed the regulations of the Ethics Committee of Affiliated Tongji Hospital of Tongji University (LL(H)-08-04 Tongji Hospital). The experimental protocol was approved by the Ethics Committee of Affiliated Tongji Hospital of Tongji University.Inclusion criteria consisted of a diagnosis of heart failure [4] and evidence of left ventricular systolic dysfunction with LVEF< 50% on 2-dimensional echocardiography obtained within 1 month prior to study. Patients with a diagnosis of significant pulmonary disease (maintained on home oxygen therapy for lung disease and/or inhaled corticosteroids) are excluded from the study.One day before CPET, treatments with digitalis, β-blocker, angiotensin converting enzyme inhibitor, angiotensin receptor blocker or diureticswere temporarily discontinued, but these treatments continued after CPET.

### Echocardiography

Echocardiography was performed on each subject using the Vivid 7 GE Echopac-system with a high-definition 3.2-MHz transducer. All data were measured by the same qualified physician. Standard M-mode and two-dimensional echocardiography and Doppler blood flow measurements were performed in agreement with the American Society of Echocardiography guidelines[5].Interventricular septum thickness (IVST) and left ventricular posterior wall thickness (LVPW) were obtained from the parasternal long axis view. The left ventricular end systolic diameter (LVESD) and left ventricular end diastolic diameter (LVEDD), were obtained from two-dimensional apical images. The LVEF was calculated from two-dimensional apical images according to the Simpson method. The left ventricular mass (LVM) was calculated according to the formula proposed by Devereux et al [6].LVM (g) = 0.8× [1.04× (IVS + LVEDD + LVPWT) ^3^—LVEDD^3^] + 0.6. LVM was subsequently adjusted for body surface area (BSA) to obtain an index: LVMI (g/m^2^) = LVM/BSA.

### CPET

Symptom-limited CPET was performed on all patients using the modified Ramp10 protocol[7] by Variobike 500 Exercise apparatus (GE, USA). All subjects were encouraged to put forth a maximal effort immediately prior to exercise testing. Ventilatory expired gas analysis was performed using a metabolic cart (Innocor version 5.00 BBB, Denmark). Before each test, the equipment was calibrated according to manufacturer’s specifications using reference and calibration gases. Standard 12-lead electrocardiograms were obtained at rest, exercise and recovery phase, blood pressure was measured using a standard cuff sphygmomanometer. Before exercising, patients were asked to rest for 3 minutes. The exercise began with a 3-min warm-up at 0 W and 60–70 rpm, and thereafter 10 W increment in load was administered every minute after exercising at 20 W for 2 minutes. CO measurements were made by the way of inert gas rebreathing by CPET at the end of the rest period, at 30 W, 60W, 90W and 120W, etc. Mean arterial pressure was calculated from the standard equation, mean arterial pressure = (systolic pressure+2*diastolic pressure)/3. Peak cardiac power output (peak CPO) was derived from the product of the peak mean arterial blood pressure and CO divided by 451 [2].Continuous ECG, manual blood pressure measurement, heart rate recording were performed every minute via the ECG, and the Borg scale was used to rate perceived exertion at each stage [8].Exercise was discontinued when patients were physically exhausted, or developed severe dyspnea or dizziness. Several parameters were obtained through the metabolic systems during exercise testing, including oxygen consumption (VO_2_), carbon dioxide output (VCO_2_), minute-ventilation (VE). The anaerobic threshold (AT) was determined by V slope [9] The VE/VCO_2_ slope was determined by using linear regression analysis of minute ventilation and carbon dioxide production obtained during the entire exercise period [10].

### Primary Endpoints

Patients were followed over median 33.7- month up to 6 years for cardiac-related mortality after CPET via medical chart review. Any death that was precipitated by cardiac dysfunction, as per the hospital discharge diagnosis, was considered to be an event. The most common causes of mortality, as per the hospital discharge diagnosis, were cardiac arrest, myocardial infarction, and end stage heart failure.

### Statistical Analysis

All continuous data were reported as mean value ± standard deviation (SD). Categorical variables were reported as percentages. Student t-test and chi-square analysis were used to compare the differences in continuous and categorical variables, respectively. NYHA grade was compared with nonparametric rank-sum test. Receiver operation characteristic analysis (ROC) and univariate analysis were employed to evaluate the predictive value of peak CPO, peak VO_2_, VE/VCO_2_ slope for cardiac-related mortality. Statistical tests with a 2-tailed *P* value of < 0.05 were considered to be significant. Subsequently, the joint effect of the explanatory variables on the time to event was examined in a multivariable analysis. A forward stepwise selection procedure was used to retain only the statistically significant variables. In the multivariate analysis, statistical tests with a 2-tailed *P* value of < 0.05 were considered to be significant. SPSS version 18.0 was used for statistical analysis.

## Results

A total of 129 patients were enrolled (113 men, 16 women) with a mean age of 59.1±11.4 years. Their body mass index (BMI) was 24.7±3.7 kg/m^2^ and LVEF was 38±9%. These patients were evaluated for their functional classes (NYHA I n = 5, NYHA II: n = 68, NYHA III: n = 56). Among them, 74 were diagnosed with coronary artery disease, and 55 had idiopathic dilated cardiomyopathy. They were currently treated with digitalis (43.0%), β-blocker (89.0%), angiotensin converting enzyme inhibitor and angiotensin II receptor blocker (91.0%) and diuretics (51.0%).

Because of technical difficulties, CO was not obtained in 21 patients (16%). Table 1 shows the characteristics of CHF patients at baseline and CPET variables. Peak VO_2_ was 14.0 ± 3.9 ml.kg ^-1^.min^-1^, VO_2_ at the anaerobic threshold was 10.5±2.4 ml. kg ^-1^.min^-1^, CO was 4.0±1.6 L/min, peak CO was 5.8±2.4 L/min, and peak CPO was 1.3±0.6W (Table 1).

**Table 1:** Clinical characteristics and CPET Variables.

n	129
Age,y	59.1±11.4
Sex (male), %	113(87.6)
Body mass index (BMI), kg/m^2^	24.7 ± 3.7
Resting mean BP, mm Hg	87.6±7.5
Resting heart rate, beats. min^-1^	71.6±5.5
Ischemic cardiomyopathy, n (%)	74(57.4)
LVEF, %	38 ± 9
LVMI, g/m^2^	138.8 ± 46.5
NYHA class, n (%)	
I	5(3.9)
II	68(52.7)
III	56(43.4)
**Medications**	
Diuretic, n (%)	66 (51.0)
β-blocker, n (%)	115 (89.0)
ACE inhibitor or ARB, n (%)	117 ((91.0)
Digoxin, n (%)	55 (43.0)
**CPET variables**	
Peak VO_2_,ml.kg^-1^.min^-1^	14.0 ± 3.9
VE/VCO_2_ slope	38.9 ± 8.7
VO_2_ at anaerobic threshold, ml.kg^-1^.min^-1^	10.5±2.4
Cardiac output,n (%), L/min	108(84),4.0±1.6
Peak cardiac output,n (%), L/min	108(84),5.8±2.4
Peak cardiac power output, n (%),Watt	108(84),1.3±0.6
Peak heart,rate, beats. min^-1^	111.6±15.5
Peak mean BP,mmHg	102.8±15.2

The characteristics of CHF patients at baseline and CPET variables. Peak VO_2_ was 14.0 ± 3.9 ml.kg ^-1^.min^-1^, VO_2_ at the anaerobic threshold was 10.5±2.4 ml. kg ^-1^.min^-1^, CO was 4.0±1.6 L/min, peak CO was 5.8±2.4 L/min, and peak CPO was 1.3±0.6W. CPET = cardiopulmonary exercise test, M = male, BMI = Body Mass Index, LVMI = Left Ventricular Mass Index, LVEF = Left Ventricular Ejection Fraction, NYHA = New York Heart Function Assessment, ACEI = Angiotensin Converting Enzyme inhibitor, ARB = Angiotensin Receptor Blocker, Peak VO_2_ = Peak Oxygen Consumption, VE/VCO_2_ slope = the minute ventilation/carbon dioxide production slope. Data are expressed as mean ± SD, unless otherwise stated.

During follow-up, 19 cardiac deaths were identified. Among those dead, there were lower LVEF, higher LVMI, lower peak VO_2_, higher VE/VCO_2_ slope, lower PeakCO and lower Peak CPO (Table 2). By using Receiver Operating Characteristic curve (ROC) analysis we were able to evaluate the predictive value of peak VO_2_,VE/VCO_2_ slope and Peak CPO for cardiac-related deaths. Their area under ROC were 0.64, 0.67, 0.68, respectively (*Ρ*<0.05). The optimal thresholds for predicting cardiac-related deaths were peak VO_2_≤13.4 ml.kg^-1^.min^-1^, VE/VCO_2_ slope≥39.3 and peak CPO≤ 1.1 W respectively (Table 3).

**Table 2:** Clinical characteristics of those dead and alive.

Variable	Total(n = 129)	Dead(n = 19)	Alive(n = 110)	*P* value
Age,y	59.1±11.4	60.7±9.3	58.8±11.8	0.505
Sex (male), %	113(87.6)	16(84.2)	97(88.2)	0.705
Body mass index (BMI), kg/m^2^	24.7 ± 3.7	23.7 ± 3.2	24.8 ± 3.8	0.238
Resting mean BP, mm Hg	87.6±7.5	86.5±6.5	87.9±5.4	0.312
Resting heart rate, beats. min^-1^	71.6±5.5	70.9±5.1	71.8±4.5	0.413
Ischemic cardiomyopathy, n (%)	74(57.4)	7(36.8)	67(60.9)	0.076
LVEF, %	38 ± 9	33 ± 9	38 ± 9	0.010
LVMI, g/m^2^	138.8 ± 46.5	158.3 ± 53.9	133.2 ± 40.1	0.028
NYHA class I/II/III	5/68/56	0/8/11	5/60/45	0.299
I,%	5(3.9)	0(0)	5(4.5)	
II,%	68(52.7)	8(42.1)	60(54.5)	
III,%	56(43.4)	11(57.9)	45(41.0)	
**Medications**				
Diuretic, n (%)	66 (51.0)	10(52.0)	56(51.0)	0.890
β-blocker, n (%)	115 (89.0)	17(89.4)	98(89.1)	0.960
ACE inhibitor or ARB, n (%)	117 ((91.0)	17(89.4)	100(90.9)	0.690
Digoxin, n(%)	55 (42.6)	8(42.1)	47(42.7)	0.901
**CPET variables**				
Peak VO_2_,ml.kg^-1^.min^-1^	14.0 ± 3.9	11.8 ± 4.3	14.4 ± 3.7	0.008
VE/VCO_2_ slope	38.9 ± 8.7	43.7.9 ±9.1	38.0 ± 8.4	0.008
VO_2_ at anaerobic threshold, ml.kg^-1^.min^-1^	10.5±2.4	9.3±3.2	10.7±2.1	0.016
Cardiac output,n (%), L/min	108(84),4.0±1.6	16(84), 3.5±1.8	92(84),4.1±1.5	0.170
Peak cardiac output,n (%), L/min	108(84),5.8±2.4	16(84), 4.5±1.8	92(84), 6.5±2.4	0.031
Peak cardiac power output, n (%),Watt	108(84),1.3±0.6	16(84), 0.9±0.2	92(84),1.5±0.5	0.007
Peak heart,rate, beats. min^-1^	111.6±15.5	105.1±18.6	112.6±14.8	0.078
Peak mean BP,mmHg	102.8±15.2	90.2±14.2	107.4±14.7	0.003

During follow-up, 19 cardiac deaths were identified. Among those dead, there were lower LVEF, higher LVMI, lower peak VO2, higher VE/VCO_2_ slope, lower Peak CO and lower Peak CPO(*P* = 0.010,0.028,0.008,0.008,0.031, and 0.007, respectively).

**Table 3:** Univariate Predictors and Area Under the Receiver Operator Characteristic Curves (ROCs) of the CPET Variables for cardiac-related deaths.

CPET variables	Hazard Ratio (95% CI)	*P* for Hazard Ratio	Optimal threshold	Area Under ROC Curve (95% CI)	*P* for Area Under ROC Curve
Peak VO_2_,ml.kg^-1^.min^-1^	2.43 (1.81–3.24)	0.02	≤13.4	0.64 (0.49,0,80)	0.045
VE/VCO_2_ slope	1.38 (1.14–2.28)	0.017	≥39.3	0.67(0.55,0.79)	0.021
Peak CPO, Watt	1.11 (1.03–1.19)	0.01	≤ 1.1 W	0.68(0.53,0.83)	0.032

By using ROC analysis, The area under ROC of peak VO_2_, VE/VCO_2_ slope and Peak CPO were 0.64, 0.67, 0.68, respectively (*Ρ*<0.05). The optimal thresholds for predicting cardiac-related deaths were peak VO_2_≤13.4 ml.kg^-1^.min^-1^, VE/VCO_2_ slope≥39.3 and peak CPO≤ 1.1 W respectively.

**Fig 1:**
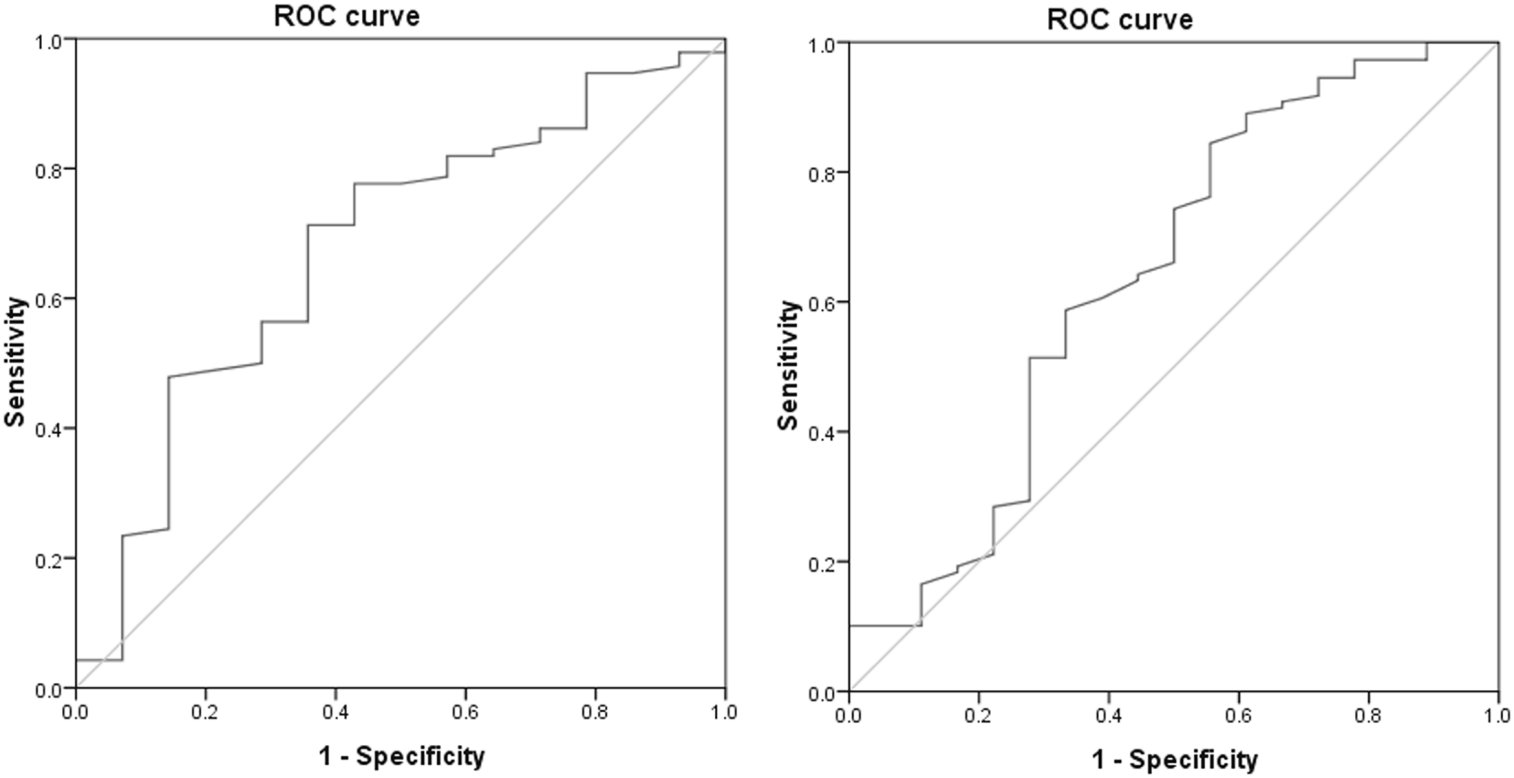
ROC analysis of peak VO_2_, Peak CPO(Left panel: peak CPO; Right panel: peak VO_2_). The area under ROC Curve of peak CPO for predicting cardiac-related deaths was 0.68 (*Ρ*<0.05), and the sensitivity and specificity were 0.713 and 0.650 respectively, which is significantly more sensitive than peak VO_2_ (the sensitivity was 0.590 and the specificity was 0.667). The optimal threshold of peak CPO for predicting cardiac-related deaths was≤ 1.1W and the optimal threshold of peak VO_2_ for predicting cardiac-related deaths was ≤13.4 ml.kg^-1^.min^-1^ in Chinese CHF patients.

**Fig 2:**
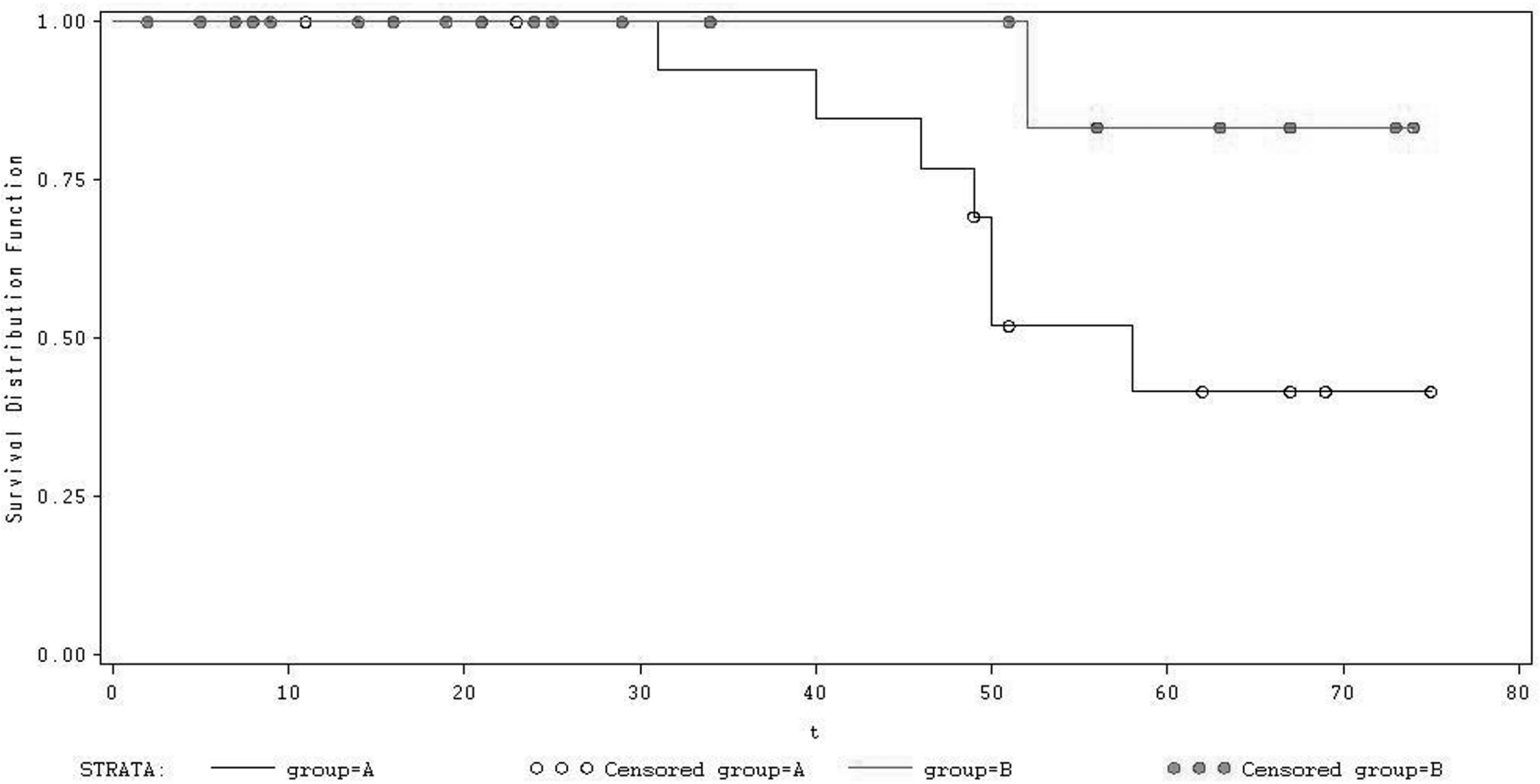
Kaplan-Meier analysis of peak CPO and peak VO_2:_Curve 1(group B): peak VO_2_≤ 13.4 ml.kg^-1^.min^-1^ but peak CPO > 1.1 W; Curve 2(group A): peak VO_2_≤ 13.4 ml.kg^-1^.min^-1^ but peak CPO ≤ 1.1W. Log-Rank: 3.875, *P* = 0.04. By Kaplan Meier analysis, the patients with a peak VO_2_≤13.4 ml.kg^-1^.min^-1^ those with peak CPO>1.1W had better survival than those with peak CPO ≤ 1.1 W (Log-Rank: 3.875, *P* = 0.04).

The area under ROC Curve of peak CPO for predicting cardiac-related deaths was 0.68 (*Ρ*<0.05), and the sensitivity and specificity were 0.713 and 0.650, which is significantly more sensitive than peak VO_2_ and VE/VCO_2_ slope. The optimal threshold of peak CPO for predicting cardiac-related deaths was≤ 1.1W and the optimal threshold of peak VO_2_ for predicting cardiac-related deaths was ≤13.4 ml.kg^-1^.min^-1^ in Chinese CHF patients (Fig 1). Moreover, by Kaplan Meier analysis we have shown that in patients with a peak VO_2_≤13.4 ml.kg^-1^.min^-1^ those with peak CPO>1.1W had better survival than those with peak CPO ≤ 1.1 W (Fig 2).

To investigate the contributions of CPET variables to cardiac-related deaths, a forward stepwise selection procedure was used to retain only the statistically significant variables. The results showed that VE/VCO_2_ slope and LVEF were predictors of cardiac-related death after adjustment for age, gender, BMI, Resting heart rate and LVMI(*P<* 0.05) (Table 4). However, peak CPO and Peak VO_2_ were not significant predictors of cardiac-related deaths(*P*> 0.05). And the Beta Values (*B*) of Peak VO_2_,VE/VCO_2_ slope,peak CPO and LVEF are -0.127(*P* = 0.099), 0.065(*P* = 0.027), -0.878(*P* = 0.242) and -0.113(*P* = 0.001), respectively (Table 4). The *B* values suggest that Peak VO_2_, peak CPO and LVEF are protective factors of cardiac-related death, while VE/VCO_2_ slope is a risk factor of cardiac-related death. The hazard ratio of above four variables are 0.880(0.757–1.024), 1.068 (1.008–1.131), 0.416(0.095–1.808) and 0.893(0.836–0.954) respectively(Table 4). After adjustment for age, gender, BMI, Resting heart rate, LVMI and LVEF, Multivariate analysis of the CPET Variables for cardiac-related deaths showed that Peak CPO, Peak VO_2_ and VE/VCO_2_ slope were not predictors of cardiac-related death (*P*> 0.05).The *B* values of Peak CPO,Peak VO_2_ and VE/VCO_2_ slope are -0.259(*P* = 0.696), -0.068(*P* = 0.352), 0.022(*P* = 0.479), the hazard ratio of the variables are 0.722(0.210–2.836), 0.934 (0.808–1.079), 1.022(0.962–1.085) respectively(Table 5).

**Table 4:** Multivariate analysis of the CPET Variables and LVEF for cardiac-related deaths adjusted for age, gender, BMI, Resting heart rate and LVMI.

Model	Variable	*B*	SE	*P* value	HR	95% CI of HR
Model 1	PeakVO_2_,ml.kg^-1^.min^-1^	-0.127	0.077	0.099	0.880	0.757–1.024
	Gender	-0.093	0.869	0.915	0.911	0.166–5.004
	Age,y	0.004	0.029	0.893	1.004	0.948–1.063
	Body mass index (BMI), kg/m^2^	-0.033	0.071	0.644	0.968	0.842–1.112
	Resting heart rate, beats. min^-1^	-0.002	0.041	0.971	0.998	0.921–1.083
	LVMI, g/m^2^	0.012	0.005	0.010	1.012	1.003–1.022
Model 2	VE/VCO_2_ slope	0.065	0.030	0.027	1.068	1.008–1.131
	Gender	0.250	0.860	0.772	1.284	0.238–6.931
	Age,y	-0.008	0.029	0.785	0.992	0.938–1.050
	Body mass index (BMI), kg/m^2^	-0.033	0.070	0.634	0.967	0.843–1.109
	Resting heart rate, beats. min^-1^	-0.008	0.039	0.838	0.992	0.920–1.070
	LVMI, g/m^2^	0.013	0.004	0.004	1.013	1.004–1.021
Model 3	Peak CPO,Watt	-0.878	0.750	0.242	0.416	0.095–1.808
	Gender	-13.91	711.685	0.984	0.000	0.000–0.000
	Age,y	0.022	0.028	0.429	1.022	0.968–1.080
	Body mass index (BMI), kg/m^2^	0.105	0.101	0.299	1.110	0.912–1.352
	Resting heart rate, beats. min^-1^	-0.033	0.053	0.534	0.968	0.873–1.073
	LVMI, g/m^2^	0.012	0.005	0.010	1.012	1.003–1.021
Model 4	LVEF(%)	-0.113	0.034	0.001	0.893	0.836–0.954
	Gender	0.176	0.850	0.836	1.192	0.225–6.309
	Age,y	0.020	0.027	0.463	1.020	0.968–1.074
	Body mass index (BMI), kg/m^2^	-0.026	0.074	0.721	0.974	0.842–1.126
	Resting heart rate, beats. min^-1^	-0.037	0.039	0.333	0.963	0.893–1.039
	LVMI, g/m^2^	0.003	0.005	0.624	1.003	0.992–1.013

Multivariate analysis of the CPET Variables and LVEF for cardiac-related deaths showed that VE/VCO_2_ slope and LVEF were predictors of cardiac-related death after adjustment for age, gender, BMI, Resting heart rate and LVMI (*P<* 0.05), However, peak CPO and Peak VO_2_ were not significant predictors of cardiac-related deaths. (*P*> 0.05). HR = Hazard Ratio.

**Table 5:** Multivariate analysis of the CPET Variables adjusted for age, gender, BMI, Resting heart rate,LVMI and LVEF.

Model	Variable	B	SE	P value	HR	95.0% CI
Model 1	Peak CPO,Watt	-0.259	0.664	0.696	0.772	0.210–2.836
	Gender	-13.541	613.883	0.982	0.000	0.000–0.000
	Age,y	0.038	0.031	0.229	1.038	0.977–1.104
	Body mass index (BMI), kg/m^2^	0.092	0.108	0.392	1.097	0.888–1.354
	Resting heart rate, beats. min^-1^	-0.048	0.049	0.333	0.953	0.865–1.05
	LVMI, g/m^2^	0.002	0.005	0.766	1.002	0.991–1.012
	LVEF(%)	-0.107	0.038	0.005	0.898	0.834–0.967
Model 2	Peak VO_2_,ml.kg^-1^.min^-1^	-0.068	0.074	0.352	0.934	0.808–1.079
	Gender	0.074	0.867	0.932	1.077	0.197–5.887
	Age,y	0.013	0.030	0.660	1.013	0.955–1.075
	Body mass index (BMI), kg/m^2^	-0.032	0.074	0.664	0.968	0.837–1.12
	Resting heart rate, beats. min^-1^	-0.029	0.038	0.451	0.972	0.901–1.047
	LVMI, g/m^2^	0.004	0.005	0.494	1.004	0.993–1.014
	LVEF(%)	-0.097	0.035	0.006	0.908	0.847–0.973
Model 3	VEVCO_2_ slope	0.022	0.031	0.479	1.022	0.962–1.085
	Gender	0.282	0.861	0.744	1.325	0.245–7.17
	Age,y	0.011	0.029	0.700	1.011	0.955–1.071
	Body mass index (BMI), kg/m^2^	-0.028	0.074	0.700	0.972	0.841–1.123
	Resting heart rate, beats. min^-1^	-0.033	0.038	0.389	0.968	0.898–1.043
	LVMI, g/m^2^	0.003	0.005	0.532	1.003	0.993–1.014
	LVEF(%)	-0.100	0.039	0.009	0.905	0.839–0.976

Multivariate analysis of the CPET Variables for cardiac-related deaths showed that Peak CPO, Peak VO_2_ and VE/VCO_2_ slope were not predictors of cardiac-related death after adjustment for age, gender, BMI, Resting heart rate, LVMI and LVEF. (*P*> 0.05). HR = Hazard Ratio.

## Discussions

The principal findings of this prospective cohort study are that peak CPO is not a predictor of cardiac death in Chinese CHF patients.

Several studies have suggested additional prognostic values of hemodynamic measurements during exercise testing in patients with heart failure. Moreover, correlations between hemodynamic data (e.g. cardiac output) and peak VO_2_ have been shown to be variable [2]. Patients with a low peakVO_2_ who might have appropriate cardiac function at peak exercise, as the etiology for the low peak VO_2_ may be the result of deconditioning, obesity or other peripheral factors. Therefore a combination of hemodynamic measurement with cardiopulmonary exercise testing is expected to greatly improve risk stratification. A low mortality risk was reported in our study that was not consisted with others studies. Several risk factors contributed to this outcome. Patients with sicker HF or elderly patients were more frequent in their studies. Additionally, female patients with more advance HF or lower LVEF contributed to these differences. Studies demonstrated that peripheral muscle mechanisms underlying advance HF concerning the fact that measurement in VO_2_ but not in CO or CPO play a more important role in reducing functional capacity and influencing mortality.

Indeed, CPO represents the rate of energy input that the systemic vasculature receives from the heart, incorporating both pressure and flow domains of the cardiovascular system [11].Therefore such a measure of cardiac pumping ability would predict outcomes for patients with cardiogenic shock and CHF, as has been demonstrated previously [12–14].Although indicator dilution technique is considered to be the gold standard for cardiac output measurement, it is invasive and expensive. In recent years, inert gas rebreathing by CPET is emerging as a novel noninvasive method to measure CO during exercise. Yet it has not been well studied in Chinese CHF population. In this study, ROC analysis for peak VO_2_ showed a moderate prognostic value. However, peak VO_2_ had no prognostic value for CHF in Chinese population by multivariable analysis.

Obviously there are interactions between peak CPO, peak VO_2_ in one side and other factors such as LVEF in another side. In univariate analysis, in patients with a peak VO_2_ of ≤13.4 ml.kg^-1^.min^-1^ those with peak CPO >1.1 W had better survival. Our data were in line with previous reports in which peak CPO was a stronger predictor than peak VO_2_ [12,15]. In terms of the optimal threshold or cut-off for predicting outcomes in CHF patients, studies are not consistent. In one study the cut-off value of peak CPO for predicting all-cause mortality was <1.96W [12].In another study the cut-off of peak CPO for predicting events (including death, urgent heart transplant, or left ventricular assist device implantation) was <1.5W in CHF patients [15]. The optimal threshold of our study was lower than above two studies. This might be due to different endpoints and different population. However, by multivariable analysis after adjustment for age, gender, BMI, Resting heart rate and LVMI, our study showed that LVEF was a significant independent predictor of cardiac-related deaths among these variables,which is consistant with other studies[16–17]. While peak CPO,VE/VCO_2_ slope and peak VO_2_ were not significant predictors of cardiac-related deaths. For the protective factors of peak VO_2_, peak CPO and LVEF, the hazard ratio of peak CPO was the smallest among these variables, which was consistant with the hypothesis about the prognostic value of peak CPO. However, peak CPO was not a significant predictor in our study possibly due to the smaller sample size of peak CPO because in 21 patients no data about peak CPO were obtained. But it still has the trend of predicting cardiac-related deaths.

### Limitations

This was a single center study with a relatively small cohort of CHF patients with a limited number of endpoints. The mortality rate in our study is 14.7% over median 33.7 months. Thus our power to detect statistical differences was low. In univariate analysis, our results demonstrated that peak CPO is statistically significantly superior to peak VO_2_≤13.4 ml.kg^-1^.min^-1^ and VE/VCO_2_ slope as a predictor of cardiac death in Chinese CHF patients as assessed by both ROC and Kaplan Meier analyses.But by multivariable analysis, peak CPO was not a significant predictor of cardiac-related deaths.

In conclusion, our results support peak CPO was not a predictor of cardiac death in Chinese CHF patients.

## Supporting Information

S1 File (XLS)

## Abbreviations

CHF: Chronic Heart Failure
CPET: Cardiopulmonary Exercise Testing
Peak VO_2_: Peak Oxygen Consumption
VE/VCO_2_ slope: theminute ventilation/carbon dioxide production slope
BMI: Body Mass Index
LVMI: Left Ventricular Mass Index
LVEF: Left Ventricular Ejection Fraction
NYHA: New York Heart Function Assessment
ACEI: Angiotensin Converting Enzyme inhibitor
ARB: Angiotensin Receptor Blocker
AT: Anaerobic Threshold
